# A Three-Surgeon–Six-Hand Operation Using a 4K-3D Exoscope for Neurological Surgery: A Case Report

**DOI:** 10.3389/fsurg.2022.866476

**Published:** 2022-03-11

**Authors:** Ryota Tamura, Yuki Kuranari, Makoto Katayama

**Affiliations:** ^1^Department of Neurosurgery, Keio University School of Medicine, Shinjuku, Japan; ^2^Department of Neurosurgery, Kawasaki Municipal Hospital, Kawasaki, Japan

**Keywords:** exoscope, 3-dimensional, 4K, KINEVO, six hand, assistant

## Abstract

**Background:**

Advances in digital imaging including evolving of 3-dimensional (3D) exoscope has allowed its use as an alternative to microscopes in neurosurgery. The exoscope can concede wide space around the operating table and patient. Here, we show a three-surgeon–six-hand operative approach using a 4K-3D exoscope. Practical advantages and disadvantages of this approach are discussed.

**Clinical Presentation:**

A 58-year-old male was refered with a 60 mm diameter meningioma in the right frontal convexity. The tumor removal was done by an operator and two assistants with a scrub nurse while viewing images displayed on a 55-inch monitor with integrated 4K and 3D visualization technology retrieved by KINEVO®. Meaningful communication between the operator and two assistants allowed for simultaneous, and precise surgical procedures. Gross total removal was achieved without damaging the brain.

**Conclusion:**

The ocular-free, openness of 4K-3D exoscope allows for a three-surgeon–six-handed operation, which leads to simultaneous surgical maneuvers by multiple hands, shorter operative time, flexible/intermittent brain retraction made by two assistants, and educational benefits owing to the surgical procedure being visually shared.

## Introduction

The incorporation of visual enhancement technology has transformed the field of neurosurgery to the next generation. The operating microscope (OM) has become the gold standard in neurosurgery (1, 2). However, the OM has some limitations in operative mobility, accessibility and expense. Additionally, the operational view of the OM is limited by the patients position, which can, in cases, leading to an uncomfortable position for the operator and assistant, resulting in intraoperative fatigue. To ameliorate these problems, the 3-dimensional (3D) extracorporeal telescope (exoscope) was created (3, 4). Owing to advances in digital imaging, the 3D exoscope has been increasingly used as an alternative to microscopes in surgery. The 4K-3D exoscope ideally presents the operative environment illustrated broadly in a 3D landscape. The exoscope is suspended above the surgical field. A 4K monitor is displayed in front of the surgeon, and the operation is performed while watching the monitor and wearing 3D glasses. A surgeon's position is not limited to the microscope' oculars, while freedom in movements during surgery, a higher comfort rate, a lower fatigue after longer procedures have been reported in using a 4K-3D exoscope (5, 6).

The exoscope system can be used for brain tumor, skull base surgery, aneurysm clipping and vascular microanastomosis, both cervical and lumbar complex spine surgery (7). In the last decade, several types of approaches using exoscopes have been developed and adapted to various neurosurgical procedures (8–11).

Similarly, endonasal endoscopic approach has evolved to enable skull base surgery through minimal access ports using pre-existing air spaces. Endoscopy provides excellent magnification, high-definition images and a panoramic view (12). Four-handed technique provides further panoramic views and greater surgical freedom with minimal invasion, and results in fewer complications compared to the two-handed technique (13).

The exoscope has a focal length of 220–650 mm (10, 14, 15). The exoscope will allow for wide space around the operating table and patient. This is especially useful in procedures of surgical assistants with multiple equipment (e.g., navigation devices or ultrasound). The purpose of this study is to show the three-surgeon–six-hand operative approach using a 4K-3D exoscope. The practical advantages and disadvantages of this approach are discussed.

## Case Presentation

### Equipment, Operating Room Setup, and Patient Positioning

KINEVO® (Carl Zeiss Meditec AG, Oberkochen, Germany) was used, which contains 4K-3D displays, light filters for 5-aminolevulinic acid and indocyanine video-angiography, pneumatic arms, adjustable operative settings, multiscreen output, longer focus distance and a greater magnification power (10, 14, 15). The operator takes position in front of the patients head with two assistants on both sides. A scrub nurse stands on the operator's dominant hand side between the operator and each assistant.

**Figure 1:**
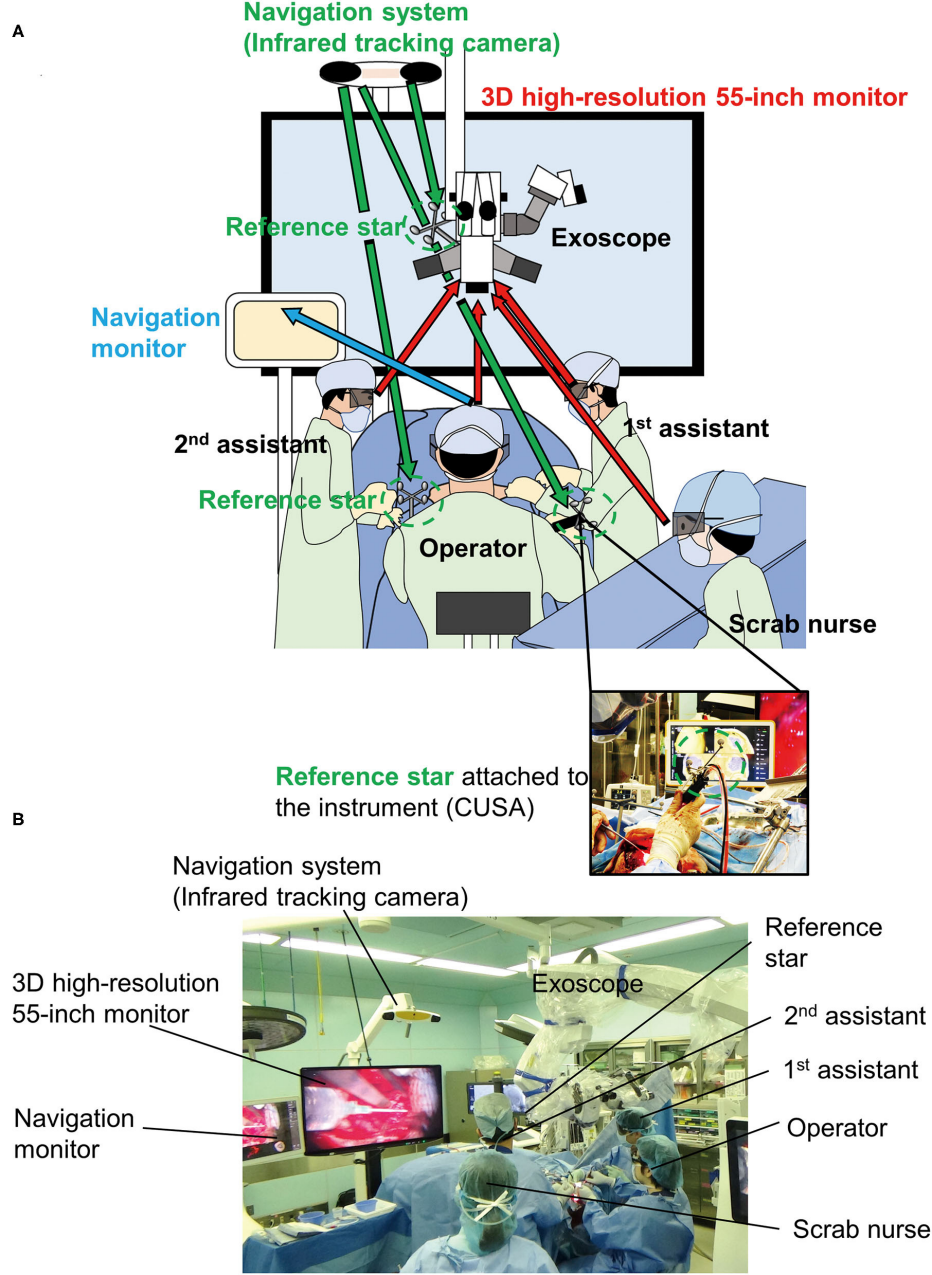
Equipment, operating room setup, and patient positioning. **(A)** Illustration of a three-surgeon–six-hand operation using a 4K-3D exoscope is shown. **(B)** Actual setup in the operation room. Arrows means viewpoint of each staff.

The KINEVO® camera was placed above the patients head at a high position allowing for open visualization of the monitor. A high-definition view of the surgical field was projected onto a 3D high-resolution 55-inch monitor, which was placed across the room toward the patient's leg side. An operator and two assistants operated with the scrub nurse viewing images (with 3D glasses) of the surgical field on the monitor. We used a neuronavigation system (BrainLab, Munich, Germany) to visualize the tumor and anatomical landmarks. Infrared tracking camera with extended detection and navigation monitor were placed behind, and next to the 55-inch monitor, respectively. The reference star was positioned around the neck caudally from the second assistant (Figures 1A,B).

### Surgical Procedures

**Figure 2:**
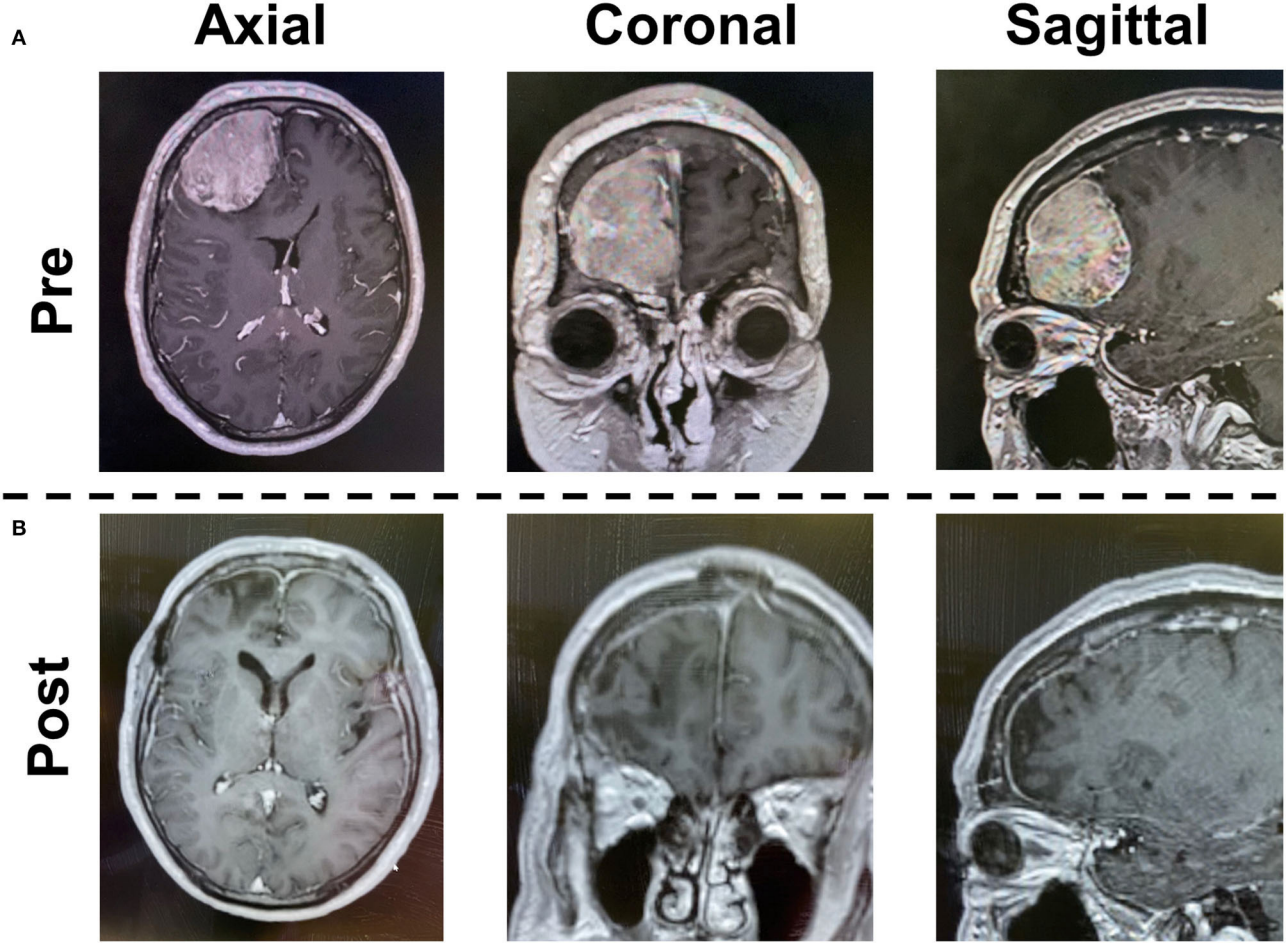
Case presentation. Preoperative **(A)** and postoperative **(B)** gadolinium-enhanced T1-weighted imagings are shown.

**Figure 3:**
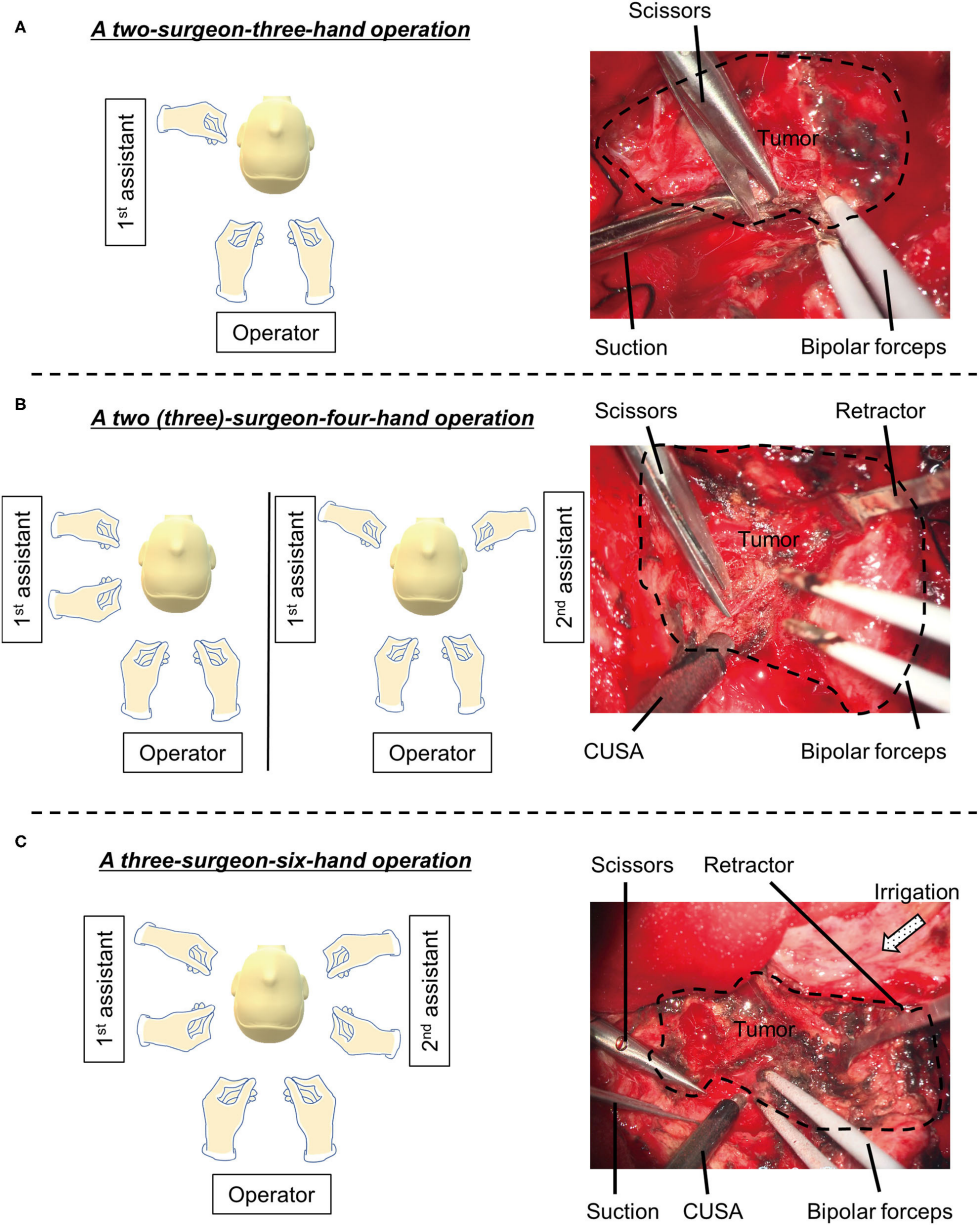
The combination of the experienced operator and assistants. A two-surgeon-three-hand **(A)**, a two (three)-surgeon-four-hand **(B)** and a three-surgeon-six-hand operations **(C)** is illustrated on the left panels. Intraoperative images are shown on the right panels.

A 58-year-old male presented with a headache. CT demonstrated a 60 mm diameter meningioma in the right frontal convexity. Tumor removal was performed *via* frontal craniotomy (Figure 2). A three-surgeon–six-hand operative technique was used with a 4K-3D exoscope, as described above (Figure 3). Furthermore, a two-surgeon-three-handed and a two (three)-surgeon-four-handed method was flexibly used (Figure 3).

During the operation, the operator mainly used bipolar forceps and CUSA® Clarity (Integra LifeSciences Corporation, NJ, USA). The first and second assistants used micro scissors and suction, or brain retractor and suction. The combination of the experienced operator and two assistants allowed for seamless processing of dissection between the brain and tumor, without exchanging tools from one hand to another. Continuous brain retraction was unnecessary. Gross total removal was achieved without damaging the brain (Simpson's grade I). Postoperative CT scan displayed complete removal of the tumor.

## Discussion

In the present study, the three-surgeon–six-hand operation was perfomed using a 4K-3D exoscope, showing some practical advantages. Exoscope has a focal length of 220–650 mm, resulting in wider working space. The camera head of the exoscope does not interfere with the assistants' access to the surgical field during simultaneous surgical procedures. It was not necessary to devise the patient's position, the operators' position, and the arrangement of other surgical equipment. Meaningful communication between the operator and two surgical assistants allowed for seamless procedures, leading to shorter operative time (Table 1).

**Table 1:** Advantages and disadvantages of the three-surgeon–six-hand operation using a 4K-3D exoscope.

**Advantage**	**Disadvantage**
1. Meaningful communication between the operator and two surgical assistants allows for seamless procedures, leading to shorter operative time. 2. No continuous brain retraction is needed. 3. Two assistants can obtain educational benefits owing to the visually and dynamically shared surgical procedures.	1. Two assistants may feel intraoperative fatigue during looking at the same monitor placed on the caudal side of the patients.

Brain retraction is important to secure surgical space during brain surgery. Surgery without continuous tumor and brain retractors could be performed because two assistants versatilely pulled the tumor or brain to the operator's desired direction. The combination of the experienced operator and assistants lead to seamless, efficient and faster dissection procedures (Table 1). With the flexible and intermittent retraction, the brain damage was morphologically minimal with a retraction force (16–18).

Typically, the use of external monitors and glasses can give the audience such as students and residents the same high-resolution view as the surgeon, leading to educational advantages (14). The three-surgeon–six-hand operation also includes special educational benefits of two assistants in addition to all individuals present in the operating room owing to the visually and dynamically shared surgical procedures. Furthermore, the operator may be attuned to surrounding trainees, leading to valuable teaching opportunities (Table 1).

An important disadvantage of this approach is the slight difficulty in assisting the operator from both sides for the two assistants, and a relatively distant position of the scrub nurse (Table 1). This disadvantage can be reduced by the efficient equipment, operating room setup, and patient positioning. Two assistants may feel intraoperative fatigue looking at the same direction having the 55-inch monitor placed on the caudal side of the patients. However, this is still better than an OM, because the assistant has to look into the other eyepiece of the OM in an uncomfortable position dictated by the primary operator.

The 4K-3D exoscope can allow wide space around the operating table and patient. We can perform the three-surgeon–six-hand operative approach. Further experience is needed to achieve more comfortable maneuverability of surgical instruments during the procedure.

## Conclusions

A three-surgeon–six-hand operation using a 4K-3D exoscope overcomes some limitations in operative mobility, accessibility and educational aspects in the OM. This approach will be further adapted for the use in various neurosurgical diseases.

## Data Availability Statement

The original contributions presented in the study are included in the article, further inquiries can be directed to the corresponding author.

## Author Contributions

RT conceptualized, designed, performed the study, and wrote the manuscript. YK assisted in the acquisition of data. MK assisted with discussion and review of the manuscript. All authors approved the final version.

## Conflict of Interest

The authors declare that the research was conducted in the absence of any commercial or financial relationships that could be construed as a potential conflict of interest.

## Publisher's Note

All claims expressed in this article are solely those of the authors and do not necessarily represent those of their affiliated organizations, or those of the publisher, the editors and the reviewers. Any product that may be evaluated in this article, or claim that may be made by its manufacturer, is not guaranteed or endorsed by the publisher.

## References

[1] Herlan S Marquardt JS Hirt B Tatagiba M Ebner FH. 3D exoscope system in neurosurgery—comparison of a standard operating microscope with a new 3D exoscope in the cadaver lab. Oper Neurosurg. (2019) 17:518–24. 10.1093/ons/opz081 31140555

[2] Siller S Zoellner C Fuetsch M Trabold R Tonn JC Zausinger S. A high-definition 3D exoscope as an alternative to the operating microscope in spinal microsurgery. J Neurosurg Spine. (2020) 33:705–14. 10.3171/2020.4.SPINE20374 32650307

[3] Khalessi AA Rahme R Rennert RC Borgas P Steinberg JA White TG. First-in-man clinical experience using a high-definition 3-dimensional exoscope system for microneurosurgery. Oper Neurosurg. (2019) 16:717–25. 10.1093/ons/opy320 30476242

[4] Pafitanis G Hadjiandreou M Alamri A Uff C Walsh D Myers S. The exoscope versus operating microscope in microvascular surgery: a simulation non-inferiority trial. Arch Plast Surg. (2020) 47:242–9. 10.5999/aps.2019.01473 32453933 PMC7264907

[5] Nishiyama K. From exoscope into the next generation. J Korean Neurosurg Soc. (2017) 60:289–93. 10.3340/jkns.2017.0202.003 28490154 PMC5426447

[6] Panchal S Yamada Y Nagatani T Watanabe T Kishida Y Sayah A. Practice survey to compare and identify the usefulness of neuroendoscope and exoscope in the current neurosurgery practice. Asian J Neurosurg. (2020) 15:601–7. 10.4103/ajns.AJNS_339_19 33145213 PMC7591211

[7] Montemurro N Scerrati A Ricciardi L Trevisi G. The exoscope in neurosurgery: an overview of the current literature of intraoperative use in brain and spine surgery. J Clin Med. (2021) 11:223. 10.3390/jcm11010223 35011964 PMC8745525

[8] Kwan K Schneider JR Du V Falting L Boockvar JA Oren J. Lessons learned using a high-definition 3-dimensional exoscope for spinal surgery. Oper Neurosurg. (2019) 16:619–25. 10.1093/ons/opy196 30124929

[9] Langer DJ White TG Schulder M Boockvar JA Labib M Lawton MT. Advances in intraoperative optics: a brief review of current exoscope platforms. Oper Neurosurg. (2020) 19:84–93. 10.1093/ons/opz276 31529083

[10] Sack J Steinberg JA Rennert RC Hatefi D Pannell JS Levy M. Initial experience using a high-definition 3-dimensional exoscope system for microneurosurgery. Oper Neurosurg. (2018) 14:395–401. 10.1093/ons/opx145 29106670

[11] Wanibuchi M Komatsu K Akiyama Y Mikami T Mikuni N. Effectiveness of the 3D monitor system for medical education during neurosurgical operation. World Neurosurg. (2018) 109:e105–9. 10.1016/j.wneu.2017.09.113 28958929

[12] Almeida JP de Albuquerque LA Dal Fabbro M Sampaio M Medina R Chacon M. Endoscopic skull base surgery: evaluation of current clinical outcomes. J Neurosurg Sci. (2019) 63:88–95. 10.23736/S0390-5616.16.03386-5 26603533

[13] Castelnuovo P Pistochini A Locatelli D. Different surgical approaches to the sellar region: focusing on the “two nostrils four hands technique”. Rhinology. (2006) 44:2–7. 16550942

[14] Ricciardi L Chaichana KL Cardia A Stifano V Rossini Z Olivi A. The exoscope in neurosurgery: an innovative “point of view”. A systematic review of the technical, surgical and educational aspects. World Neurosurg. (2019) 124:136–44. 10.1016/j.wneu.2018.12.202 30660891

[15] Vetrano IG Acerbi F Falco J D'Ammando A Devigili G Nazzi V. High-definition 4K 3D exoscope (ORBEYETM) in peripheral nerve sheath tumor surgery: a preliminary, explorative, pilot study. Oper Neurosurg. (2020) 19:480–8. 10.1093/ons/opaa090 32357216

[16] Ahmad FI Mericli AF DeFazio MV Chang EI Hanasono MM Pederson WC. Application of the ORBEYE three-dimensional exoscope for microsurgical procedures. Microsurgery. (2019) 40:468–72. 10.1002/micr.30547 31855291

[17] Kanzaki S Takahashi S Toda M Yoshida K Ogawa K. Pros and cons of the exoscope for otologic surgery. Surg Innov. (2021) 28:360–5. 10.1177/1553350620964151 32990502

[18] Yokoh A Sugita K Kobayashi S. Intermittent versus continuous brain retraction. An experimental study. J Neurosurg. (1983) 58:918–23. 10.3171/jns.1983.58.6.0918 6854385

